# A cynomolgus monkey *E. coli* urinary tract infection model confirms efficacy of new FimH vaccine candidates

**DOI:** 10.1128/iai.00169-24

**Published:** 2024-09-19

**Authors:** Laurent Chorro, Tara Ciolino, Caresse Lynn Torres, Arthur Illenberger, JohnPaul Aglione, Paula Corts, Jacqueline Lypowy, Christopher Ponce, Annalena La Porte, Deborah Burt, Gretchen L. Volberg, Lila Ramaiah, Kathryn McGovern, Jianfang Hu, Annaliesa S. Anderson, Natalie C. Silmon de Monerri, Isis Kanevsky, Robert G. K. Donald

**Affiliations:** 1 Pfizer Vaccine Research and Development, Pearl River, New York, USA; 2 Pfizer Drug Safety Research and Development, Groton, Connecticut, USA; 3 Pfizer Drug Safety Research and Development, Pearl River, New York, USA; 4 Pfizer Research Biostatistics, Collegeville, Pennsylvania, USA; University of California Davis, Davis, California, USA

**Keywords:** UTI, non-human primate, cystitis, UPEC, *Escherichia coli*, FimH

## Abstract

The increase in urinary tract infections (UTI) caused by antibiotic-resistant *Escherichia coli* requires the development of new therapeutic agents and prophylactic vaccines. To evaluate the efficacy of new lead candidates, we implemented a cynomolgus macaque UTI challenge model that mimics human uncomplicated cystitis in response to transurethral challenge with a multidrug-resistant (MDR) *E. coli* serotype O25b ST131 isolate. *E. coli* fimbrial adhesin FimH and O-antigens are separately under clinical evaluation by others as vaccine candidates to prevent UTI and invasive urosepsis disease, respectively. Accordingly, we assessed the protective efficacy of three 50-µg intramuscular doses of a novel recombinant FimH antigen adjuvanted with liposomal QS21/MPLA compared with saline placebo in groups of nine animals. A third group was vaccinated with this FimH formulation in combination with 1 µg each of a four-valent mixture of serotype O1a, O2, O6, and O25b O-antigen CRM_197_ lattice glycoconjugates. Both vaccines elicited high levels of serum FimH IgG and adhesin blocking antibodies at the time of bacterial challenge and, for the combination group, O-antigen-specific antibodies. Following bacterial challenge, both vaccinated groups showed >200- and >700-fold reduction in bacteriuria at day 2 and day 7 post-infection compared with placebo, respectively. In parallel, both vaccines significantly reduced levels of inflammatory biomarkers IL-8 and myeloperoxidase in the urine at day 2 post-infection relative to placebo. Results provide preclinical proof-of-concept for the prevention of an MDR UTI infection by these new vaccine formulations.

## INTRODUCTION

Urinary tract infections (UTI) are among the most common bacterial infections worldwide. It is estimated that 50% of women will experience at least one UTI in their lifetime (1, 2). While most UTI present as uncomplicated urinary bladder inflammation (cystitis), bacteria can also ascend from the bladder to the kidneys, resulting in pyelonephritis. Pyelonephritis can lead to severe renal tissue damage and more serious manifestations such as bacteremia and sepsis, which require emergency medical treatment, though these complications are less common in healthy, non-diabetic adults (3, 4). While multiple bacterial species can be responsible for UTI, Uropathogenic *Escherichia coli* (UPEC) is the predominant cause of uncomplicated and complicated cystitis (3, 5–7). UPEC is also responsible for 80% of acute pyelonephritis cases in women and 70% of cases in men (8). *E. coli* sequence type 131 (ST131) has recently emerged globally as a dominant multidrug-resistant (MDR) extraintestinal pathogenic *E. coli* (ExPEC) responsible for UTI and other forms of invasive disease including sepsis (9). *E. coli* ST131 strains are mostly of the O25b:H4 serotype. A recent analysis of globally sourced *E. coli* UTI isolates from the Antimicrobial Testing Leadership and Surveillance (ATLAS; https://atlas-surveillance.com/) program revealed that ST131 O25b was the predominant O-antigen serotype in U.S. bloodstream infections and in patients with bladder, kidney, ureter, and urethra infections (10). The ST131 O25b clone is highly virulent in the urinary tract, and infections are frequently community associated (11, 12). Additionally, most *E. coli* ST131 are resistant to aminopenicillin and fluoroquinolone antibiotics (10, 13–15), limiting treatment options for this clonal lineage.

UPEC typically originate in the gut (7, 16, 17), contaminating the urogenital tract by adhering to host uroepithelial cells and then replicating rapidly once reaching the permissive bladder niche. Adhesion is facilitated by the type 1 fimbrial adhesin FimH, which binds to mannosylated glycoproteins expressed on the surface of uroepithelial cells (18). UPEC FimH null mutants show reduced ability to establish bladder infections in murine and porcine models of UTI (19–22) and fail to form intracellular bacterial communities in a human urothelial microtissue model of infection (23). FimH has been preclinically validated as a vaccine antigen to prevent UTI, by eliciting antibodies that neutralize the binding of bacteria to mannoside receptors (22, 24). Similarly, small molecule antagonists that block ligand binding have been developed as therapeutic agents for UTI treatment (25–27).

There are currently no licensed vaccines for the prevention of UTI or therapeutic maintenance of recurrent UTI, although a FimH candidate has completed a phase 1 clinical study, and a second O-antigen vaccine candidate preventing urosepsis in the elderly population has progressed to phase 3 (ExPEC9V) (28, 29). Preclinical studies evaluating the efficacy of candidate UPEC-targeted vaccines are supported by animal models, including small rodents, pigs, and primates (22, 24, 30–37). Cynomolgus macaques represent a promising animal model, as UPEC in this species causes an infection that closely resembles uncomplicated cystitis cases observed in women. The mean duration of bacteriuria in these non-human primates (NHPs) ranged from 7 to 30 days, depending on the UPEC strain used for challenge, after which the infection cleared naturally within an average of 3 weeks (22, 33, 38–43). The immune response elicited by UPEC in NHPs has yet to be described in the context of a preclinical NHP UTI challenge model, but urine inflammatory biomarkers associated with UTI in humans include interleukin (IL)-6, IL-8, and myeloperoxidase (MPO) (44–47). Signs of protection against UPEC-induced UTI by a first FimH vaccine candidate were initially demonstrated in a Cynomolgus macaque model using qualitative methods to assess reduction in pyuria and bacterial colonization (22).

Here, we developed an acute cystitis model in female cynomolgus macaques by challenging animals with a contemporary serotype O25b ST131 UPEC isolate. Our model captures two key features of human infection: (i) a sustained and robust bacteriuria upon infection and (ii) the recruitment and activation of innate immune cells and detection of IL-8 and MPO biomarkers. We describe the use of this updated UTI challenge model to evaluate the protective efficacy of two exploratory FimH vaccine antigen formulations.

## MATERIALS AND METHODS

### Bacterial strain and stock preparation

The *E. coli* ST131 O25b clinical isolate PFEEC0578 was selected from the Pfizer-sponsored ATLAS collection [https://atlas-surveillance.com/; maintained by the International Health Management Associates (IHMA)] for NHP inoculation. The strain was collected in 2017 from the bladder of a 38-year-old male. It carries genes that encode an unknown type of capsular polysaccharide and is MDR. Based on results of *in vitro* antibiotic susceptibility testing, it is resistant to levofloxacin and amoxicillin/clavulanic acid (amoxicillin-clavulanic acid). The genome sequence of PFEEC0578 is available as an NCBI Sequence Read Archive (BioProject no. PRJNA804716, Accession no. SAMN26379143). This strain and derived FimH mutant are described elsewhere as UPEC strain EC10 (23). Bacterial stock was prepared by inoculating 12 mL of lysogeny broth (LB) (Teknova), incubating overnight at 37°C with agitation at 275 rpm. The culture was then diluted 1:10 in LB and incubated for 2–3 hours at 37°C and mixed with 25 mL of glycerol (80%, MP) for long-term storage. The concentration of viable bacteria was confirmed by plating serial dilutions on TSA plates [BD, BBL Trypticase Soy Agar (Soybean Casein Digest Agar)] and analysis after 18 hours of incubation at 30°C.

### Animals

Female cynomolgus macaques (*Macaca fascicularis*) were obtained from Charles River Laboratories (Houston, TX) (age range: 4–5 years, weight range: 3.1–5.9 kg). NHPs were housed in standard quad caging with water and food provided *ad libitum*. Animals were microchipped subcutaneously to monitor internal temperature. NHPs free of *E. coli* infection were selected based on negative urine quantitative PCR (qPCR) results. Studies were performed at Pfizer, Pearl River, NY, which is accredited by the Association for Assessment and Accreditation of Laboratory Animal Care (AAALAC). All procedures performed on these macaques were in accordance with regulations and established guidelines and were reviewed and approved by an Institutional Animal Care and Use Committee.

### NHP vaccines and immunization schedules

FimH-DSG, the FimH vaccine antigen used in this study, is a full-length FimH protein based on the protein sequence from *E. coli* strain J96. It is produced in mammalian cells and harbors mutations to eliminate non-native N-glycosylation (a consequence of heterologous expression of FimH in mammalian cells) (N7S, N70S, and N228S), mutations to constrain conformation (V27A, G15, and G16), and a stabilizing C-terminal Donor Strand G peptide (DSG) (48, 49). Vaccine antigens were adjuvanted with AS01_B_ (50 µg of MPL and 50 µg of QS-21, GSK). O1a, O2, O6, and O25b O-antigen CRM_197_ glycoconjugates were constructed as described previously for serotypes O25b and O1a (10, 50). Doses were calculated based on Anthrone saccharide concentration for each individual conjugate. Cynomolgus macaques were immunized intramuscularly (IM) (0.55 mL) at weeks 0, 4 and 14 either with vehicle control [phosphate buffered saline (PBS), pH 6.2] or FimH-DSG antigen (50 µg/dose) or with a mixture of four-valent O25b, O1a, O2, and O6 O-antigen polysaccharide CRM_197_ conjugates (1 µg/dose) in combination with FimH-DSG (50 µg/dose). On weeks 0, 6, and 16, 10 mL of blood was collected via the femoral vein into one serum separator tube (BD Vacutainer), using a 21G safety needle/vacutainer. Collection tubes were left at room temperature for 30 min and centrifuged at 3,000 × *g* for 10 min. Serum in the supernatant was collected, aliquoted, and stored at −80°C.

### FimH whole cell neutralization assays

*E. coli* CFT073 (ATCC) was serially passaged in 10 mL of static LB cultures to enrich for FimH expression. Surface expression reaching >95% was confirmed via flow cytometry using an anti-FimH mAb (mAb 926, reconstructed from patent sequences) (51). Prior to the assay, 384 well white Maxisorp plates (Nunc) were coated with 20 µg/mL of yeast mannan (Sigma-Aldrich) and blocked in 1% BSA (Thermo). FimH expressing *E. coli* cells were then incubated with serial dilutions of vaccinated NHP sera and controls. Sera were diluted in PBS + 0.1% BSA and titrated 2.5-fold for seven points, starting at 1:100. After 45 min at 37°C, the mixture was added to the plate and incubated for 45 min at 37°C before washing away any unbound bacteria. A titration of anti-FimH_LD_ rabbit sera was used as an internal control on every plate. Specificity of bacterial binding to mannan was established by the inclusion of Methyl α-D-mannopyranoside (Sigma) as a positive control, which reduced binding by >95% at 50 mM levels. Adherent cells were measured with a luminescent probe BacTiter Glo (Promega) and read on a Clariostar Plus plate reader. Half-maximal inhibitory concentration (IC_50_) inhibition values were interpolated using sigmoidal dose response variable-slope curve fitting (GraphPad Prism). Titers are the reciprocal of the serum dilution at which half-maximal inhibition is observed. Responders were defined as those with >50% inhibition at the starting dilution and had a defined IC_50_, a positive hillslope, *r*^2^ > 0.80, and at least two points trending toward neutralization. The limit of detection (LOD) is designated as a dilution of 50, which is half of the maximum dilution. The statistical significance (*P* value) of differences in responses between groups was determined using an unpaired *t*-test with Welch’s correction applied to log-transformed data.

### FimH IgG direct binding Luminex immunoassay

FimH-DSG antigen was coupled to spectrally distinct MagPlex-C microspheres (Luminex), which were diluted in blocking buffer to the concentration 50,000 beads/mL for 1–2 hours at room temperature while shaking immediately prior to assay primary incubation. The diluted microsphere mixture was added to assay plates containing appropriately diluted NHP serum samples, controls, and the reference standard, a humanized in-house monoclonal antibody (mAb FimH Y202) that binds the pilin domain of FimH-DSG, for incubation overnight at 2°C–8°C while shaking. After washing off non-bound components, a purified R-Phycoerythrin goat anti-human IgG, Fcγ fragment-specific secondary antibody (Jackson ImmunoResearch Laboratories) was added to the microsphere mixture and incubated for 90 min at room temperature while shaking. The magnitude of the fluorescent phycoerythrin (PE) signal measured by a Luminex FLEXMAP 3D reader was directly proportional to the amount of anti-FimH-DSG IgG bound to the protein-coupled microspheres. The data were analyzed using a custom SAS application, which uses a log/log linear regression model of the standard curve to interpolate antigen-specific antibody concentrations (µg/mL) from median fluorescent intensity. A lower limit of quantitation (LLOQ) of 0.763 µg/mL was calculated from standard curve bias. For comparative analysis of NHP urine and serum samples, hyperimmune polyclonal serum sourced from FimH-immunized NHPs was used to replace the FimH mAb as the internal standard for interpolating IgG titers. In this case, FimH polyclonal antibody stocks of arbitrary concentration assignment (U/mL) were prepared in serum and urine devoid of FimH-specific IgG to mimic sample conditions within respective assays and to normalize data across matrices.

### O-Antigen IgG dLIA

*E. coli* O-antigen polysaccharides of serotype O25b, O1, O2, and O6 were conjugated to poly-L-lysine and then coupled to MagPlex-C microspheres (Luminex) with a standard EDC/NHS coupling protocol. Microspheres were incubated with serially diluted NHP serum samples, controls, and polyclonal standards overnight at 2°C–8°C while shaking. Bound serotype-specific IgG was detected with PE-conjugated goat anti-human IgG. Fcγ fragment-specific secondary antibody (Jackson ImmunoResearch Laboratories) and fluorescence were measured by a Luminex 200 reader (Bio-Rad Laboratories). Human serum containing IgG to all four O-antigens was used as an internal standard for interpolating NHP serum IgG concentrations. An arbitrary value of 100 U/mL was assigned for each antigen-specific IgG of the standard, which yielded comparable slope profiles and signal output. LLOQ was defined as 2 U/mL.

### Serum interleukin IL-1β, IL-6, and tumor necrosis factor alpha

IL-1β, IL-6, and tumor necrosis factor alpha (TNF-α) were simultaneously quantified in thawed serum samples using the Millipore Milliplex MAP Non-Human Primate Cytokine Magnetic Bead Panel reagent kit (PRCYTOMAG-40K). The kit utilizes a multiplex immunoassay with fluorescently coded magnetic beads coated with a specific capture antibody (MagPlex-C microspheres) and Luminex xMAP (microbead-based) technology for quantifying cytokines. Briefly, cytokine standards or cytokines in serum samples were captured by beads, and then, a biotinylated secondary antibody was added, followed by a streptavidin-PE conjugate. After incubation and washing, the beads were quantified based on the fluorescent reporter signals using the Bioplex Luminex 200 instrument. LLOQ for the cytokine assays is reported as follows: <3.2 pg/mL for IL-1β and IL-6 and <16.0 pg/mL for TNF-α.

### C-Reactive protein assay

The Atellica CH High-Sensitivity C-Reactive Protein (hsCRP) assay is for *in vitro* quantitative determination of the concentration of C-reactive protein (CRP) in human serum and plasma (lithium heparin, potassium EDTA) using the Atellica CH Analyzer. The Atellica CH hsCRP latex reagent is a suspension of uniform polystyrene latex particles coated with anti-CRP antibody. When serum or plasma containing CRP is mixed with the latex reagent, agglutination takes place resulting in an increase in turbidity. This turbidity is measured at 571/658 nm. The CRP concentration in serum or plasma is determined from a calibration curve that is generated with the calibrators.

### Anesthesia and intravesical inoculation

NHPs were anesthetized with a ketamine/Dexdomitor injection via IM route (at 6 mg/kg and 0.05 mg/kg, respectively). Prior to urinary catheterization, NHPs had their anogenital area aseptically cleaned with sterile saline and benzalkonium wipes. A sterile 5 French rubber catheter coated with Surgilube surgical lubricant was inserted into the urethra and passed through to the bladder, and urine was collected. 1 × 10^8^ CFU of *E. coli* PFEEC0578 was then administered through the catheter along with 1 mL of sterile saline. Sentinel animals were given sterile saline only. The catheter was removed, and the animals were held laterally until recovery from anesthesia. NHPs received an anesthetic reversal injection of 0.5 mg/kg Antisedan (Zoetis).

### Urine sample collection and monitoring of the infection

Animals were monitored daily for up to 30 days post-infection for clinical symptoms, including urine output, behavior/appetite changes, pain/discomfort, and body temperature. Clean urine samples were collected by catheterizing the bladders of anesthetized NHPs as described above. After catheter placement, urine was voided by natural flow or aspiration. If bladders were empty, 10 mL of saline was infused and aspiration through the catheter was repeated. All samples were stored on ice immediately.

### Whole blood collection, white blood cell, and neutrophil counts

Using a 21G safety needle, 5.5 mL of whole blood was collected from the femoral vein into K2 EDTA anticoagulated vacutainer tubes. Immunogenicity bleed timepoints included weeks 0, 1, 2, 3, 4, 5, 6, 7, 9, 11, 14, and 16. Challenge was initiated at week 19 with sampling at days 0, 2, 7, 14, 21, and 28. The white blood cell (WBC) and neutrophil counts were obtained using the ADVIA 2120i hematology system (Siemens). The WBC count was obtained utilizing the basophil/lobularity method. EDTA-anticoagulated whole blood was mixed with the ADVIA 2120i Baso reagent (Siemens) causing red blood cell and platelet lysis. The WBC were stripped of their cytoplasm through a combination of the BASO reagent and an increased temperature in the reaction chamber (32°C–34°C). The cells then passed through the flow cell where two angle light scatter signals were used to quantify the number of WBC. Neutrophil counts were obtained using a peroxidase cytochemical reaction. EDTA-anticoagulated whole blood samples were mixed with ADVIA 2120i Peroxide 1 reagent (Siemens) where the surfactants in the reagent combined with thermal stress lysed the erythrocytes. The remaining leukocytes were fixed with formaldehyde and then mixed with 4-chloro-1-naphthol and hydrogen peroxide for staining of peroxidase activity. The cells passed through the flow cell where the forward light-scattering signature and absorption of each blood cell were measured and displayed in two histograms, the PEROX Y histogram containing the forward-scattering data (cell size) and the PEROX X histogram containing the absorption data (peroxidase staining) to form the Perox cytogram from which cells were identified and counted.

### Bacterial DNA extraction and quantitation

*E. coli* DNA was extracted from up to three replicates of NHP urine samples using Qiagen MinElute Kits with modifications: 500 µL starting volume, 500 µL buffer AL, 50 µL proteinase K, and 37°C molecular grade water (Corning) in place of elution buffer EB. Columns were incubated for 5 min before the final spin. Bacterial load of *E. coli* O25b in extracted samples was measured by qPCR using primers TTGAAAGTGATGGTTTGGTAAGAAAT and TGCAGCACGTATGATAACTTCAAAG and a Fam-florescence labeled probe AGGATATTTTACCCAGCAGTGCCCCGT targeting the *orf10* region. Primers and probe were reconstituted in Tris-EDTA (TE) buffer at 100 nmol/mL. DNA samples were assayed in 96-well plates with a total volume of 25 µL per well, using 12.5 µL Taqman Fast Advanced Master Mix (Thermo Fisher Scientific), 0.125 µL of each primer, 0.5 µL of probe, 1.75 µL molecular grade water, and 10 µL of sample. qPCR was run using the following cycle conditions: 50°C for 2 min, 95°C for 2 min, and 40 cycles of 95°C for 3 sec and 60°C for 30 secs on either a 7500 or Quant studio 6 real-time PCR system (Applied Biosystems). A standard curve for quantification of bacteria by qPCR was created using serial dilutions of *E. coli* from 1 × 10^9^ to 10 CFU/mL in sterile PBS and urine matrix, confirmed by plating on agar and counting colonies. DNA extraction was done using the same method as above, with standards run on every assay plate to allow interpolation of test sample CFU values by linear regression analysis (Quant Studio Software). Standard curves were linear between 100 and 1 × 10^8^ CFU/mL, with an LLOQ of 100 CFU/mL.

### Urine MPO ELISA

MPO levels in NHP urine samples were measured using a Myeloperoxidase Instant Enzyme-Linked ImmunoSorbent Assay (ELISA) Kit (Invitrogen). Samples were mixed with PIPES buffer (5% final, Alfa Aesar) and assayed in duplicate using the manufacturer’s protocol. Results were analyzed using SpectraMax Plus spectrophotometer (Danaher) at 450 nm and analyzed using SoftMax Pro software.

### Urine IL-8 Luminex assay

Levels of IL-8 in urine were measured using a custom Luminex assay kit (Bio-Rad Laboratories). Urine samples were mixed with PIPES buffer (5% final, Alfa Aesar), vortexed, and diluted before being assayed in duplicate according to the manufacturer’s protocol. Results were read on the BioPlex 200 Luminex instrument (Bio-Rad Laboratories) and analyzed using BioPlex Manager software. Standards included in the kit were used to extrapolate sample concentrations from fluorescence intensity and to determine the LLOQ.

### Microscopic analysis of urine sediments

Urine aliquots were fixed with formalin within an hour of collection, stored at 4°C, and processed the next day. Microscopic analysis of urine sediment was performed using KOVA Glasstic slides. 6.6 µL of urine was drawn into the slide chamber by capillary action resulting in a homogenous suspension of urine sediment. The formed elements of the urine were then quantified. An aliquot of urine sample was used to prepare a cytospin slide. Samples were loaded into a Shandon EZ double cyto funnel (Thermo Fisher Scientific) and centrifuged using a CytoSpin 4 cytocentrifuge (Thermo Scientific) at 750 RPM for 5 min. Slides were stained with Giemsa and May-Grunwald stain using the SP-10 automated stainer (Sysmex) and evaluated for the presence of polymorphonuclear (PMN) cells by light microscopy. The presence of increased PMN was determined relative to the background epithelial cell population.

### Statistical analyses

All statistical analyses were performed on log-transformed data. The statistical significance (*P* value) of differences in serum IgG or functional responses between groups was determined using an unpaired *t*-test with Welch’s correction. Significance was defined as *P* < 0.05. Analogous protective efficacy comparisons were also assessed on log-transformed data. For IL-8, baseline adjustment was applied due to different baseline levels in different animals. For viable counts and qPCR, baseline adjustment was not applied since all animals had the same baseline levels. The differences in viable counts, qPCR, and IL-8 among all study groups were compared using analysis of variance on each day post-vaccination. Furthermore, pairwise comparison of each vaccinated group to the placebo group was performed. Multiple comparison from the pairwise testing was adjusted by Dunnett’s test to control for alpha level. Pearson correlation between vaccine-induced antibodies (serum neutralization titer, serum IgG, and urine IgG) and UTI biomarkers (bacteriuria, IL-8, and MPO) on day two and day seven post-challenge was evaluated on the combined data from the three groups (placebo, FimH, and FimH plus O-Ag). By combining data from all three groups, the expanded data ranges allow detection of potential correlations in the context of variable responses among a limited number of animals.

## RESULTS

### Challenge with an *E. coli* O25b ST131 isolate establishes an acute, self-resolving UTI in cynomolgus macaques

UPEC ST131 serotype O25b isolate PFEEC0578 was selected for use as the bacterial challenge strain for development of a NHP model of UTI based on a combination of phenotypic and genotypic criteria. Antibiotic profiling showed that it is resistant to fluoroquinolone and aminopenicillin antibiotics. The genetic virulence factor profile of this strain closely matches that of the reference ST-131 O25b UPEC isolate EC958 (52). Like EC958, it harbors genes for iron acquisition proteins *iha*, *chuA*, *iuCC*, and *iutA* and the secreted toxin genes *sat* and *pic*. Both EC958 and PFEEC0578 lack genes for the PapGII adhesin and the *hylA* toxin. Selection of isolate PFEEC0578 as an appropriate representative of UPEC in this model was corroborated based on phenotypic screening in an *in vitro* human bladder urothelial microtissue model (23). Along with other UPEC evaluated, this isolate [also named EC10 (23)] caused umbrella cell permeability, cytotoxicity, and exfoliation that was not observed among commensal bladder isolates or an asymptomatic bladder bacteriuria strain (HM50). The isogenic PFEEC0578 *fimH* mutant had significantly impaired invasion capabilities and failed to form intracellular bacterial communities (IBCs).

**Fig 1:**
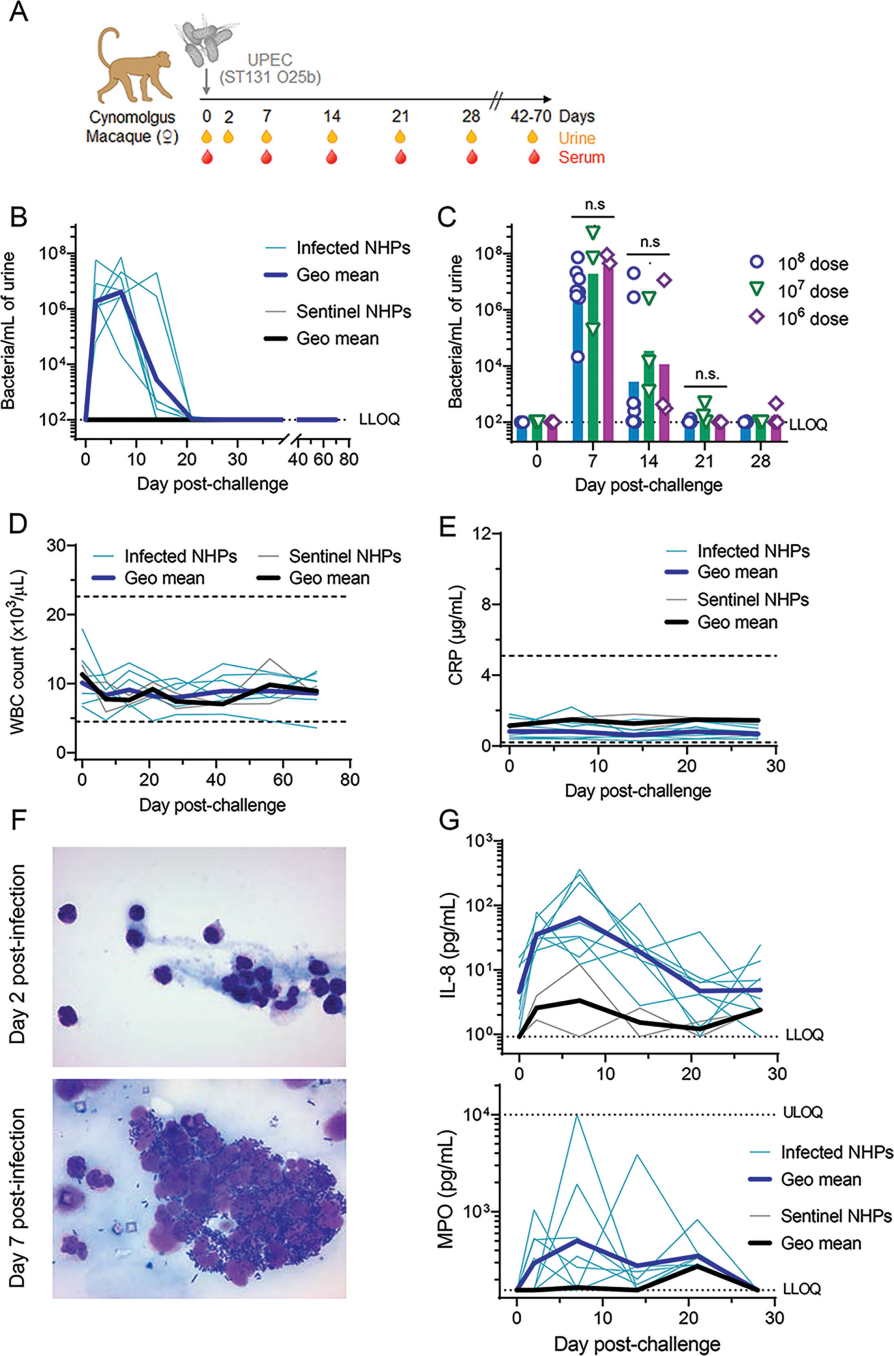
Cynomolgus macaque model of UTI challenge. (**A**) Timeline of blood draws and urine sampling by catheterization following transurethral challenge with *E. coli* ST131 O25b bacteria. (**B**) Time course of bacteriuria after administration of 1 × 10^8^ bacteria via intravesical catheterization on day 0. Thin lines represent individual animals, and thick lines indicate geometric means within each group. Dotted lines indicate the LLOQ. (**C**) Resolution of induced bacteriuria in cynomolgus macaques infected with different challenge doses: 1 × 10^8^ (A, *n* = 8; blue bars), 1 × 10^7^ (B, *n* = 3, green bars), or 1 × 10^6^ (C, *n* = 3, purple bars) viable *E. coli*. (**D and E**) No systemic signs of infection were observed in NHPs inoculated with O25b *E. coli*. WBC count and serum CRP. Dotted lines represent the reference range of values in healthy animals (CRP: 0.2–5.1 µg/mL); thin lines represent individual animals, and thick lines indicate geometric means within each group. (**F and G**) Recruitment of PMN cells and detectable levels of IL-8 and MPO in urine confirms bladder inflammation in response to the inoculation of *E. coli*. Light microscopy micrographs of urine sediments collected at days 2 and 7 post-challenge. Urine samples collected via catheterization were cytospun into slides and stained with Giemsa. Representative images show the presence of PMN cells in urine sediment of infected animals. Concentration of IL-8 and MPO in urine over a period of 28 days. Thin lines represent individual animals, and thick lines indicate geometric means within each group. Dotted lines represent the LLOQ.

Nine female cynomolgus macaques were inoculated via bladder catheterization with 1 × 10^8^ CFU of the bacterial challenge isolate. As controls, two sentinel cynomolgus macaques were infused with saline. The infection course was determined by measuring the bacterial load in urine over 70 days (Fig. 1A through C). To eliminate the possible contamination of urine samples by feces harboring the *E. coli* strain used for challenge, a sampling method of aseptic urine collection via bladder catheterization was implemented. Bacteriuria was quantified by qPCR using probes and primers specific for *E. coli* strains of the O25b serotype, including the MDR isolate used in this study. In infected animals, a high bacterial load was observed as soon as day 2 (~1 × 10^6^ bacteria/mL) and peaked at day 7 post-infection (~4 × 10^6^ bacteria/mL) (Fig. 1B). No spikes in bacterial load were observed in catheter-collected urine beyond 21 days, indicating resolution of UTI in all infected animals. Additionally, the impact of challenge dose on both magnitude and duration of bacteriuria was assessed. The bladders of macaques were infused with either 1 × 10^7^ or 1 × 10^6^ CFU of *E. coli* (day 0), and bacteriuria was monitored for up to 28 days. Compared with animals infected with 1 × 10^8^ CFU, lowering the infection dose 10- or 100-fold had no significant impact on the magnitude of bacteriuria at days 7, 14, and 21 post-infection (Fig. 1C). All animals reached peak bacteriuria at day 7 post-infection (geometric mean ~ 2–6 × 10^7^ bacteria/mL), and by day 14, the quantity of bacteria recovered in the urine had decreased, with no detectable levels by day 28 post-infection. Dilutions of urine samples were also cultured on erythromycin selection plates as an alternative method to enumerate viable bacteria in infected animals with results closely mirroring those of the qPCR-based quantification method (data not shown). To evaluate the reproducibility of the model, three additional independent challenge studies using the 1 × 10^8^ inoculum were performed, which confirmed the results of the primary study (Fig. S1A).

Next, we evaluated whether bladder instillation of the serotype O25b ST131 UPEC challenge strain resulted in systemic inflammation. Compared with uninfected controls, all challenged animals showed no variation in either rectal or microchip temperatures during the first 14 days after challenge with values remaining stable after clearance of the infection (Fig. S1B). In addition, no increase in peripheral WBC or neutrophils was observed in infected NHPs (Fig. 1D; Fig. S1C). Serum CRP levels remained consistent at <2 µg/mL throughout the course of infection at similar levels to those observed in the sentinels (Fig. 1E). Similarly, serum IL-1β, IL-6, and TNF-α levels in infected animals were all measured at low concentrations and found within their respective normal ranges for cynomolgus macaques (Fig. S1D, dashed lines). Whether vaccinated or not, none of the NHPs challenged with the ST-131 O25b UPEC isolate displayed any signs of pain or discomfort and passed normal stool and urine throughout the study.

Finally, we measured the infiltration of PMNs to the bladder by analyzing urine sediments and quantifying associated biomarkers. Urine samples obtained from the sentinel animals remained virtually free of PMNs throughout the study whereas infected macaques revealed an abundance of PMNs on day 2 and day 7 post-infection (Fig. 1F). To quantify PMN-associated local inflammatory responses, levels of IL-8 and MPO were measured in urine over the course of infection. By day 2 post-infection, both IL-8 and MPO were readily detectable in the urine of challenged macaques with a peak observed at day 7 and concentrations progressively decreasing at subsequent timepoints before reaching baseline levels at day 28 post-infection (Fig. 1G). Notably, the kinetics of both PMN recruitment and IL-8 and MPO levels mirror the kinetics of bacteriuria.

In summary, instillation of the *E. coli* ST131 O25b clinical isolate induces a robust and reproducible bacteriuria that lasted for 7 to 14 days (~ 10^6^–10^8^ bacteria/mL) that was not associated with signs of pyelonephritis or bacteremia (e.g., pyrexia) and resolved without antibiotic intervention within 3 weeks post-challenge. The recruitment of PMN cells and the presence of elevated levels of IL-8 and MPO in urine support the occurrence of bladder inflammation in this model.

### Vaccination with FimH-DSG elicits potent total and ligand binding blocking antibodies in NHPs

**Fig 2:**
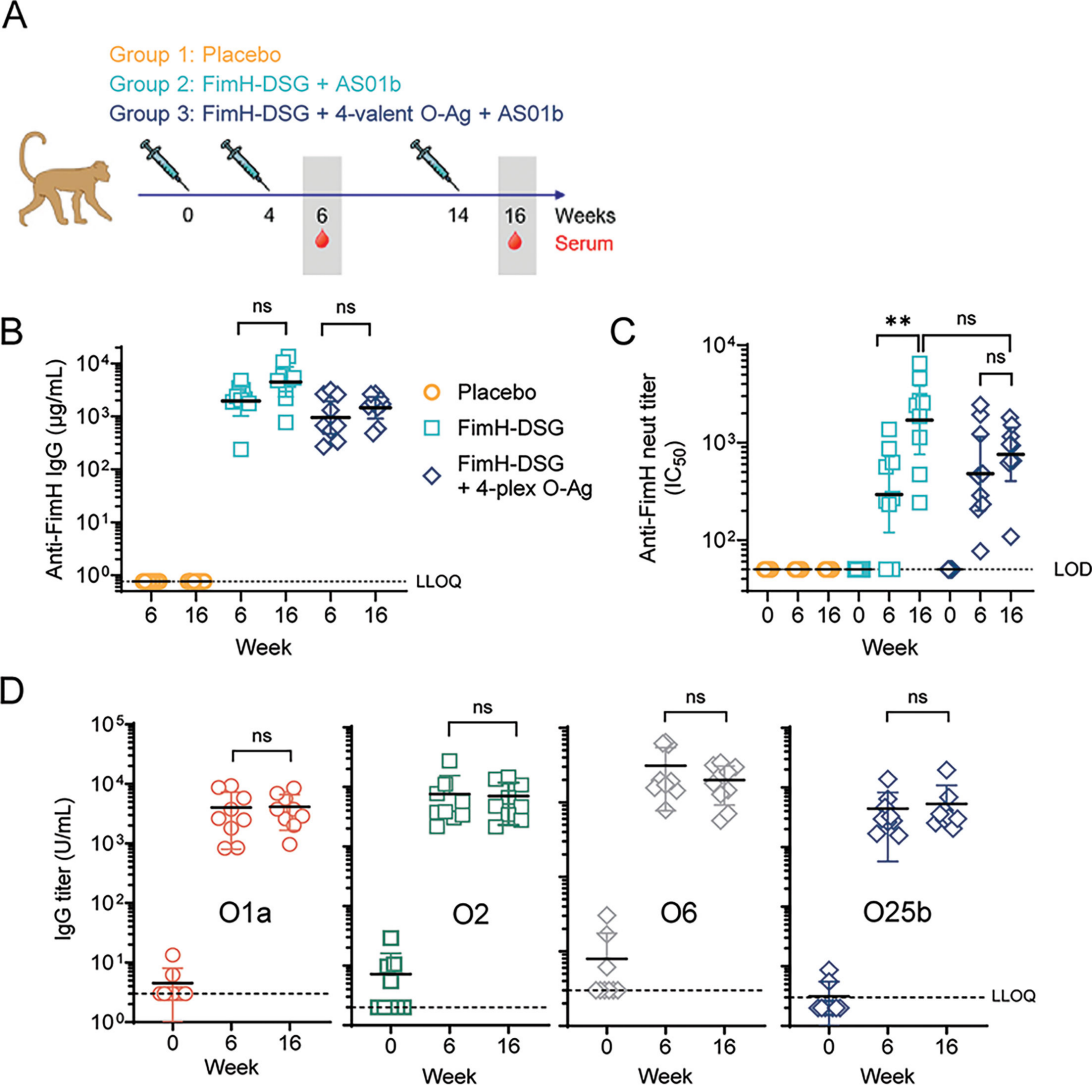
Vaccine immunogenicity. (**A**) Schedule of NHP vaccinations, bleeds prior to UPEC challenge (*n* = 1 experiment with nine NHPs/group). NHPs were immunized with placebo (gray), adjuvanted FimH-DSG alone or in combination with a tetravalent O-antigen conjugate mixture at weeks 0, 4, and 14. (**B**) Graph shows serum anti-FimH IgG titers measured at post-doses 2 and 3. Each symbol represents an individual animal. Error bars indicate geometric mean with 95% CI. Dotted line indicates the LLOQ. (**C**) Live cell ligand binding inhibition titers (log IC_50_) in yeast mannan assay at pre-vaccination and at post-dose 2 and post-dose 3 time points. (**D**) Anti-O1a, O2, O6, and O25b IgG titers in the sera of NHPs immunized with FimH-DSG in combination with a tetravalent O-antigen conjugate mixture at pre-vaccination (week 0) and at post-doses 2 (week 6) and 3 (week 16).

We designed a subunit protein antigen referred to as FimH-DSG, composed of the *E. coli* FimH lectin and pilin domains complemented by a C-terminal donor-strand peptide from FimG (48, 49). The lectin domain harbors two mutations, G15A and G16A, locking the N-terminal mannose binding pocket into an open conformation that eliminates ligand binding activity while retaining functional immunogenicity. Two groups of nine cynomolgus macaques were vaccinated three times at weeks 0, 4, and 14 with either 50 µg of FimH-DSG alone or with a combination of FimH-DSG and a mixture of 1 µg each of four O-antigen CRM_197_ glycoconjugates (serotypes O25b, O1a, O2, and O6) (10, 50). Both antigen preparations were adjuvanted with the liposomal QS21 formulation AS01_B_ (Fig. 2A). As a control, an additional group of nine cynomolgus macaques received PBS as placebo. Anti-FimH IgG titers were quantified using a direct binding Luminex immunoassay assay (Fig. 2B; Table S1). Animals in the placebo group had titers at or below the limit of quantification in this assay. In both vaccinated groups, serum anti-FimH IgG titers rose after two doses and were enhanced by a third dose (although the increase was not significant). Next, sera were evaluated in an *E. coli* adhesin blocking (or neutralization) assay to assess the ability of vaccine-elicited anti-FimH antibodies to inhibit binding of live *E. coli* to yeast mannan (Fig. 2C; Table S2). The geometric mean IC_50_ of inhibitory sera from animals vaccinated with FimH-DSG alone rose to 294 post-dose 2 and 1,698 post-dose 3 (5.8-fold increase). Similarly, the mean IC_50_ of sera from animals vaccinated with FimH-DSG antigen combined with the tetravalent mixture of O-antigen increased to 480 post-dose 2 and 756 post-dose 3 (1.6-fold increase). Finally, we also quantified the levels of O-antigen IgG in the sera of animals vaccinated with the four-valent O-antigen/FimH formulation (Fig. 2D; Table S3). After two immunizations, IgG levels against all four O-antigen serotypes rose approximately 1,000-fold relative to pre-vaccination levels. The third vaccination did not further enhance O-antigen-specific IgG levels in the group of animals vaccinated with the combination FimH and four-valent O-antigen vaccine. Together, these data show that both the FimH-DSG antigen and the FimH-DSG/four-valent O-antigen conjugate formulations elicit robust serum anti-FimH IgG and functional antibody responses in NHPs.

### Vaccination with FimH-DSG reduces biomarkers of infection in the NHP cystitis model

**Fig 3:**
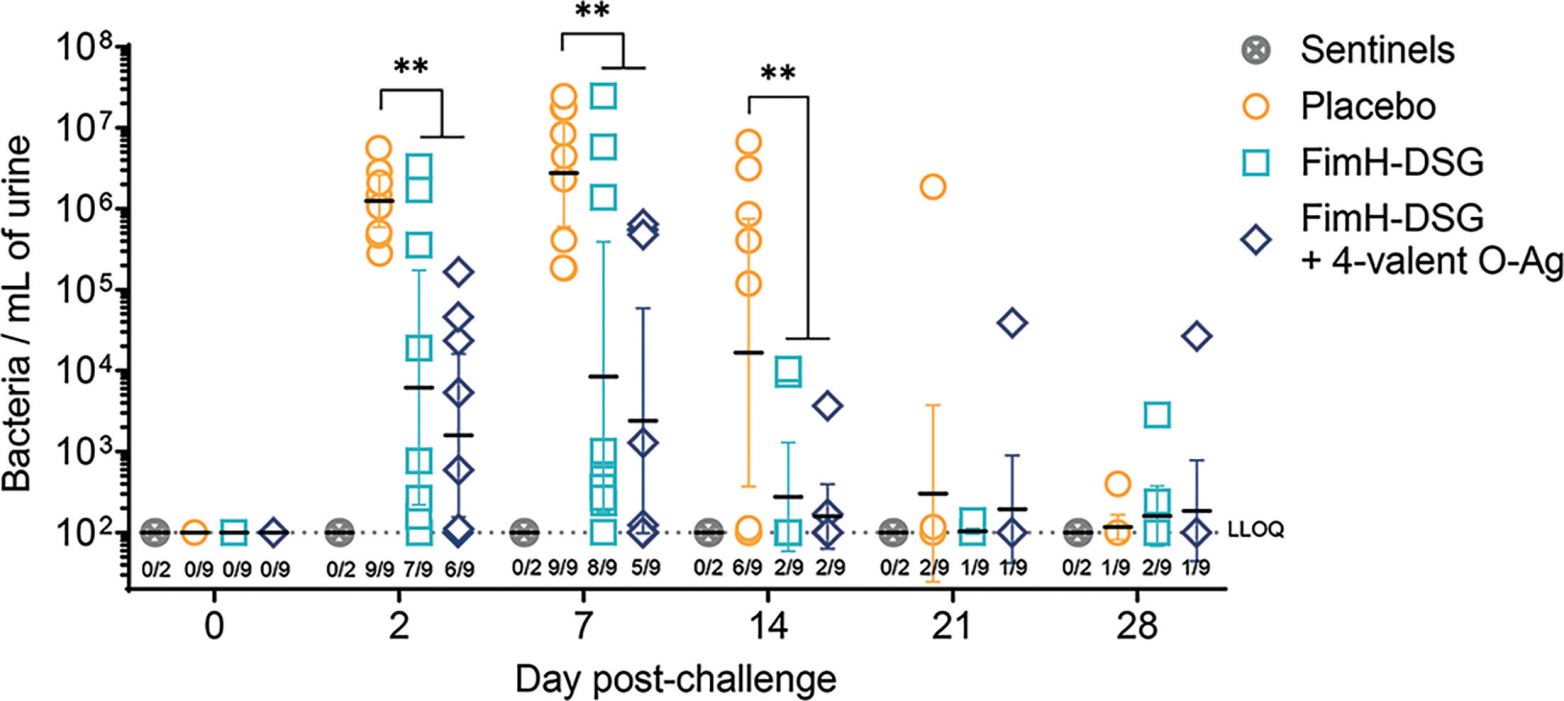
Bacteriuria measured by qPCR over 28 days. NHPs were immunized with adjuvanted FimH-DSG alone or in combination with a tetravalent O-antigen conjugate mixture at weeks 0, 4, and 14 (Fig. 2). On week 19, 10^8^ viable UPEC (*n* = 9) or PBS (*n* = 2; sentinels, open symbols) were administered via intravesical catheterization. On indicated days post-challenge, urine samples were collected via bladder catheterization. Each symbol represents an individual animal. Ratios indicate the numbers of NHPs with urine sample presenting >100 bacteria/mL over the total number of challenged animals. Error bars indicate geometric mean with 95% CI. Dotted line indicates the LLOQ.

Five weeks after the final boost (or 19 weeks after the first vaccination), vaccinated and placebo-treated NHPs were inoculated via intravesical catheterization with 10^8^ CFU of the ST131 O25b serotype challenge strain. Bacteriuria was monitored in urine collected via a catheter over a period of 28 days (Fig. 3). In all placebo-treated animals, the instillation of live bacteria led to a high level of bacteriuria on days 2 and 7 post-challenge (geometric mean of approximatively 10^6^ bacteria/mL of urine at day 2 and day 7, respectively). Compared with the placebo group, animals vaccinated with both formulations exhibited a significant reduction in bacteriuria as early as day 2 post-infection (>200- and >700-fold reduction at day 2 and day 7 post-infection, respectively). On day 14, more than half of placebo-vaccinated animals still exhibited bacteriuria > 10^5^ bacteria/mL of urine, with nearly 90% of placebo NHPs clearing the infection by days 21 and 28. In contrast, most vaccinated animals had already cleared the infection by day 14 (Fig. 3).

**Fig 4:**
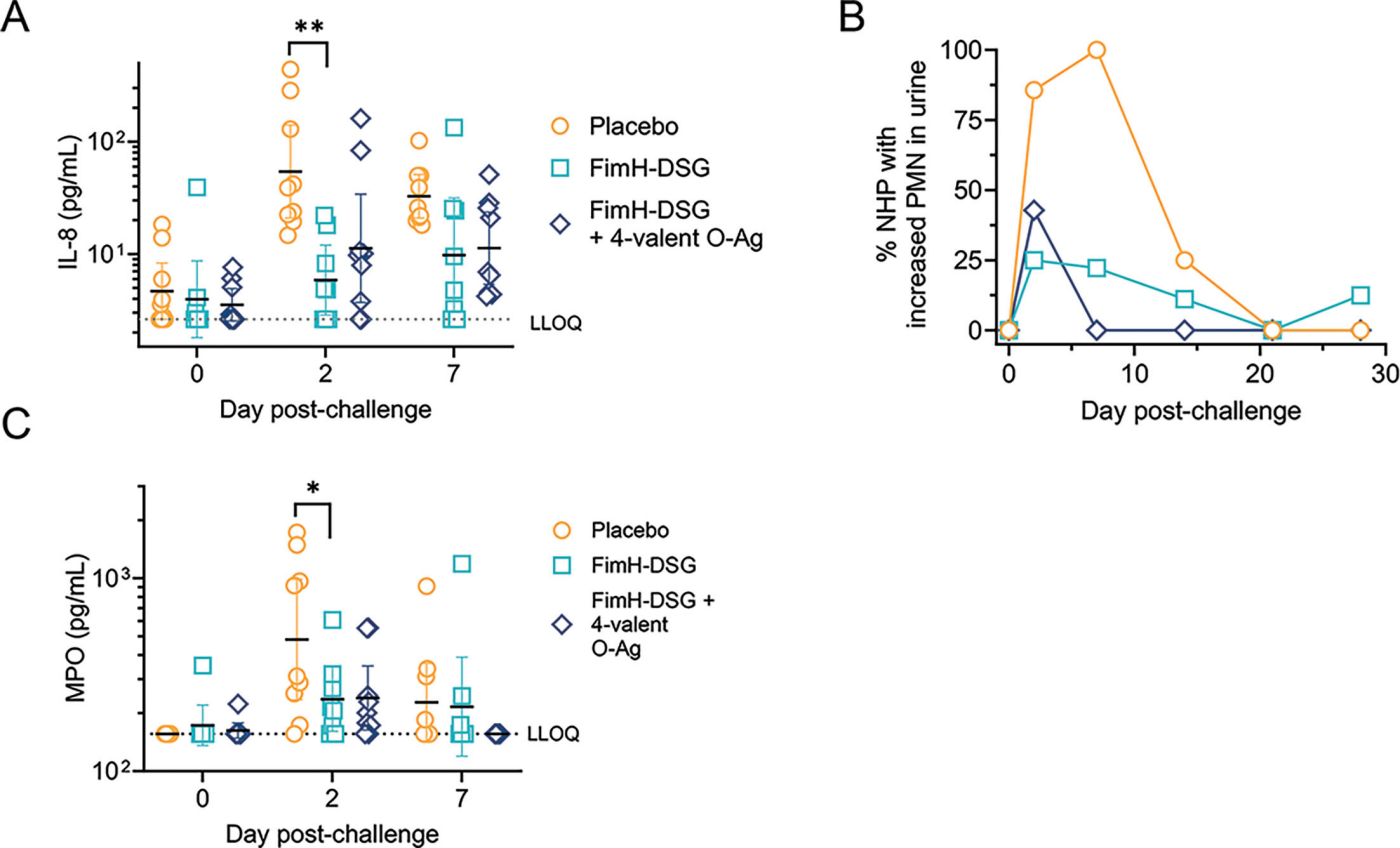
Vaccine efficacy. NHPs (*n* = 9) were immunized with placebo, adjuvanted FimH-DSG antigen alone or in combination with a tetravalent O-antigen conjugate mixture at weeks 0, 4, and 14. On week 19, 1 × 10^8^ viable UPEC isolate was administered via intravesical catheterization. (**A**) Concentration of IL-8 in urine of individual NHPs over a period of 7 days post-challenge. Error bars indicates geometric mean with 95% CI. Dotted lines represent the LLOQ. (**B**) Percentage of NHPs with PMN cell increase in urine over a period of 28 days post-challenge. On indicated days post-challenge, urine samples collected via bladder catheterization were cytospun into slides and stained with Giemsa and May-Grunwald stain. Slides were evaluated by light microscopy for the presence or absence of increased PMN cells. (**C**) Concentration of MPO in urine during the post-challenge period (as in panel A).

Next, various inflammatory biomarkers were assessed in the urine of challenged NHPs. We started by measuring the levels of chemokine IL-8 over a 7-day period because of its essential role in the recruitment of neutrophils into the bladder. On day 2 post-infection, urine concentrations of IL-8 in the FimH-DSG and combination vaccine groups had decreased by approximatively 10- and 3.5-fold, respectively (geometric mean of 5.9 pg/mL and 11 pg/mL) compared with levels measured in the urine of placebo-treated NHPs (geometric mean of 54.2 pg/mL) (Fig. 4A). On day 7 post-infection, both the FimH-DSG and combination vaccine groups exhibited an approximately threefold reduction of urine IL-8 concentration compared with unvaccinated animals (geometric mean of approximately 10 pg/mL and 33 pg/mL in vaccinated and placebo-treated NHPs). As expected, the elevated levels of IL-8 in the urine of the placebo group were consistent with the presence of PMNs in the urine sediments of all unvaccinated animals as confirmed by cytology analysis (Fig. 4A and B). In contrast, less than 25% of FimH-DSG-vaccinated NHPs and none of the FimH-DSG and tetravalent O-antigen conjugate combination-vaccinated animals had increased levels of PMN cells in urine sediment, correlating with the reduced levels of urine IL-8 observed in these animals (Fig. 4A and B). Finally, we measured urine levels of MPO, an enzyme secreted by activated PMN cells, over a 7-day period. On day 2 post-challenge, both groups of vaccinated animals exhibited a significant twofold reduction in MPO levels (geometric mean of ~200 pg/mL) compared with the placebo group (geometric mean of 480 pg/mL) (Fig. 4C). In contrast, by day 7 post-infection, urine MPO levels were close to baseline and comparable between groups.

### Analysis of vaccine-induced FimH antibody levels at the time of challenge and UTI biomarker responses

To explore the potential link between vaccine-induced antibodies and protective efficacy in this model, we compared FimH serum functional assay titers and FimH serum or urine IgG titers at the time of transurethral challenge (19 weeks after the first vaccine dose) with UTI biomarker responses at day 2 and day 7 post-challenge. Serum FimH live-cell ligand-binding inhibition GMT titers of either the FimH subunit or FimH O-antigen combination vaccine groups were approximately threefold lower than at the earlier week 16 (two weeks post-dose 3) timepoint, but differences in GMT between the groups were not significant (Fig. S2A and B). A modified anti-FimH IgG assay was implemented to allow direct comparison of total FimH IgG titers in both serum and urine samples, based on an internal polyclonal antibody standard. Compared with the unvaccinated placebo group, FimH urine IgG titers at week 19 were significantly increased in response to three doses of either of the two FimH vaccine formulations, although they were 10^3^–10^4^-fold lower than the corresponding serum IgG titers (Fig. S2C and D; Table S4). Correlation analysis confirmed an inverse relationship between FimH antibody titers from all three assays and post-challenge UTI biomarker responses. Linear regression plots and correlation value summaries are shown in Fig. S3; Table S5. Statistically significant correlations were observed between FimH urine, FimH serum IgG, or FimH serum functional antibodies at the time of challenge and UTI bacteriuria or IL-8 responses at day 2 and day 7 post-challenge. The weaker correlation observed between FimH antibody titers and MPO levels is likely due to the low sensitivity of the MPO ELISA assay, with approximately half of the samples with reportable MPO levels at the LOD of the assay (Table S4).

A fourth vaccine dose given at week 52 boosted FimH urine IgG or functional antibody responses in both vaccine groups to similar levels. Meanwhile, analogous FimH antibody titers in the unvaccinated placebo group challenged with the O25b UPEC serotype remained at or close to the limit of assay detection. These results indicate that unlike the FimH vaccine formulations, the challenge infection alone does not elicit detectable FimH antibodies.

## DISCUSSION

Much of our understanding of UPEC infection *in vivo* has come from decades of research in murine models (53). However, mice are not naturally susceptible to UPEC and require large inocula to establish a persistent non-ascending UTI relative to the small size of the bladder (54, 55). In addition, infected mice exhibit key differences in UPEC adhesin and fimbriae gene expression (56–59), as well as copper mobilization (33) as compared with UTI in humans or NHPs. Host inflammation and infection kinetics can also vary greatly depending on the mouse strain used (60). Such limitations warrant the need to evaluate large-animal infection models that better resemble the anatomy and physiology of humans. More recently, pigs have been proposed as an animal model of UTI as an alternative to the conventional mouse model (19, 35). A common limitation of all animal challenge models is that the relatively high dose required to establish a UTI does not mimic natural UTI infections, which arise in the bladder from a relatively low number of bacteria ascending from colonized urethral or peri-urethral surfaces. We observed that a relatively high-dose transurethral inoculum of 10^6^–10^8^ CFU is required to ensure model reproducibility, presumably to allow sufficient bacteria to induce FimH and other relevant UPEC virulence factors (required for attachment and invasion) prior to loss from the bladder through urination. Despite these shortcomings, the NHP UTI challenge model may represent an expeditious method for vetting new lead drug or vaccine candidates prior to advancing them into larger scale clinical safety and immunogenicity studies.

Use of cynomolgus macaques to model UPEC cystitis and test therapeutics has not been reported in the literature in over two decades, although a recent study utilized vervet monkeys (33). These older studies shared several pitfalls, including a qualitative measure of inflammation, where leukocyte excretion was visibly graded on color reactions in Ecur^4^-Test sticks dipped in urine (22, 39, 41). Our present study utilized quantitative assays to measure MPO and IL-8 levels in urine post-infection and thus provides an updated, precise, less subjective measure of local bladder inflammation. The *E. coli* strains used in these previous studies involved serotypes O6 or O4 or serotypes of unknown origin (22, 39, 41). Our study utilizes a human UPEC isolate of the most prevalent ST131 O25b serotype associated with MDR UTI (10). Bladder infusion reproducibly induced sustained bacterial colonization for up to 3 weeks post-inoculation, after which the infection resolved in the absence of antibiotics. Infection with the *E. coli* challenge strain was not associated with signs of bacteremia (pyrexia, leukocytosis), which is consistent with clinical cystitis and previously reported NHP models of cystitis (39, 41). The absence of acute phase response cytokines, such as TNF-α and IL-6, in the blood is consistent with earlier reports from children with UTI (61). Significant bacteriuria alone is insufficient to define UTI, as bacteriuria may represent an asymptomatic *E. coli* colonization of the bladder (62). UTI are clinically associated with symptoms linked to inflammation of the bladder that stems from the activation of inflammatory mediators and recruitment of immune cells (63). In this study, the peak bacteriuric period (days 5–15 post-infection) was associated with a visible increase of PMN cells in urine sediments and higher levels of neutrophil-associated inflammatory biomarkers IL-8 and MPO in urine compared with uninfected sentinels. This inflammatory response confirmed that the bacteriuria observed in this model was indeed the result of an acute bladder infection (cystitis).

An initial UTI vaccine candidate comprised of FimH in complex with periplasmic chaperone FimC showed signs of efficacy in cynomolgus macaques and was subsequently evaluated in a small Phase 1 clinical study with the TLR4 ligand monophosphoryl lipid A (MPL) adjuvant (22, 28). Although no conclusions could be made about the clinical efficacy of this FimCH vaccine, no serious safety issues were encountered (28). In the cynomolgus macaque model described here, we evaluated an alternative FimH antigen candidate, FimH-DSG, that was rationally designed to improve immunogenicity and bioprocess scalability in mammalian cells (48, 49). The AS01_B_ adjuvant in the formulation includes the QS21 saponin and an MPL component, which work synergistically to enhance vaccine immunogenicity (64). Following the pioneering work of the original FimCH NHP study by Langermann et al. (22), we confirmed herein measurable efficacy of this novel FimH-DSG formulation in reducing bladder bacteriuria and inflammation in this revised model.

*E. coli* O-antigens are preclinically validated for their ability to elicit production of opsonophagocytic antibodies capable of preventing systemic invasive disease (10, 50, 65, 66), but the extent to which they contribute to protection against UTI requires further investigation. The recent results of a phase 1b trial evaluating the ExPEC4V vaccine, a four-valent *E. coli* O-antigen conjugate vaccine targeting the O1a, O2, O6, and O25b serotypes, did not support a role of anti-O-antigen antibodies in protection against cystitis in women with history of recurrent UTI (67). While we did not evaluate the efficacy of a four-valent O-antigen glycoconjugate alone in our cynomolgus macaque study, the FimH-DSG plus O-antigen glycoconjugate combination did not significantly interfere with FimH immunogenicity or efficacy, which could prove beneficial in the context of a vaccine to prevent urosepsis.

To this day, antibiotics remain the only approved treatment option for UTI, and currently, there is no licensed vaccine to prevent UPEC infections in the United States, United Kingdom, or Europe. However, sublingual mucosal vaccines such as Uromune (largely used off-license) that utilize inactivated bacteria have shown encouraging signs of efficacy in preventing UTI (68), although the treatment regimen and crudity of the formulation may limit its application. The high incidence and recurrence rate of UTI caused by *E. coli*, along with the rapid rise of multidrug resistance, necessitate the development of new licensed antibiotic therapies and prophylactic vaccines. Moreover, use of relevant animal models is crucial to translate new antibiotic therapies and vaccine candidates into the clinic, in addition to furthering our understanding of UPEC pathogenesis and mechanisms of protection. This study builds on previous cynomolgus macaque UTI models of cystitis by employing quantitative assays to monitor bacteriuria and the inflammatory response to subsequent infection, as well use of a contemporary MDR *E. coli* O25b:H4 ST131 isolate as challenge strain. Additionally, our improved model recapitulates the time course of the bladder inflammatory response observed in untreated women with cystitis (45–47). Significantly, this study provides preliminary evidence that vaccine-induced FimH antibodies in serum and urine, at the time of UPEC challenge, are inversely correlated with UTI bacteriuria and inflammatory biomarkers. Future studies will benefit from utilization of a more sensitive MPO assay and an expanded number of vaccinated subjects, which can perhaps best be addressed in a dedicated clinical efficacy study. Having confirmed the protective efficacy of new formulations of FimH antigen in preventing UTI, we hope that larger clinical studies will identify relevant assay correlates of vaccine protection while increasing our understanding of the underlying disease mechanisms.

## SUPPLEMENTAL MATERIAL

Supplemental material iai.00169-24-s0001.pdf Fig. S1 to S3; Tables S1 to S5.

ASM does not own the copyrights to Supplemental Material that may be linked to, or accessed through, an article. The authors have granted ASM a non-exclusive, world-wide license to publish the Supplemental Material files. Please contact the corresponding author directly for reuse.
