# Supplementary material for: A cynomolgus monkey E. coli urinary tract infection model confirms efficacy of new FimH vaccine candidates
Source: Infect Immun. 2024 Sep 19;92(10):e00169-24. doi: 10.1128/iai.00169-24 (PMC11475676; doi:10.1128/iai.00169-24)
Supplement: Supplemental material — Fig. S1 to S3; Tables S1 to S5. [file iai.00169-24-s0001.pdf]

SUPPLEMENTAL FIGURE 1

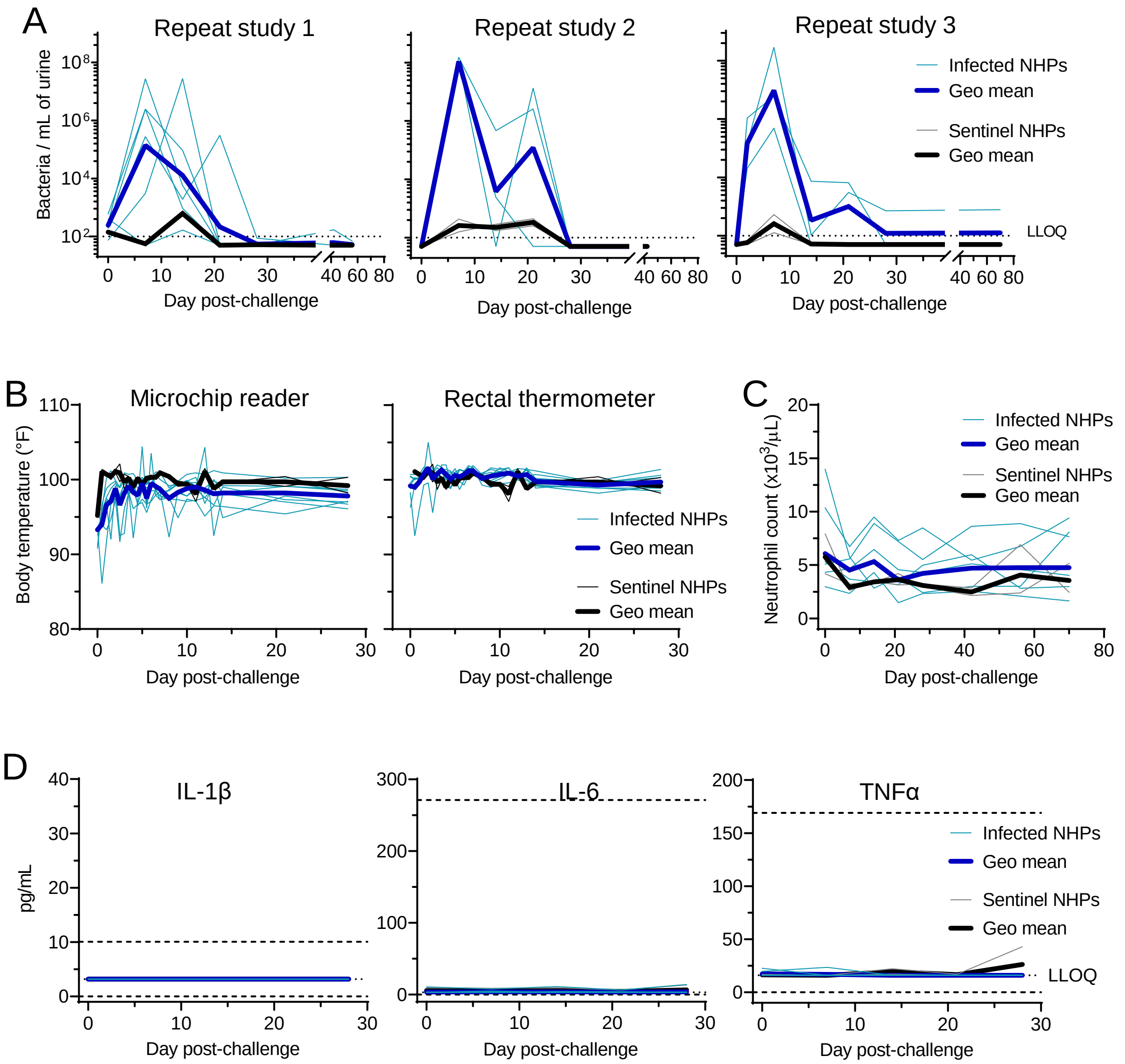

Fig. S1. UPEC challenge studies. (A) $1x10^8$ viable *E. coli* (n=8; blue line) or PBS (n=2; black line) were administered via intravesical catheterization on day 0. Urine samples were collected as in Fig. 1A. Levels of bacteriuria over time was determined as the number of CFU per mL of urine measured by plating serially diluted urine samples on erythromycin TSA plates. $1x10^8$ viable *E. coli* (blue line, n=6, repeat study 1 or n=3, repeat study 2 and 3) or PBS (n=2; black line) were administered via intravesical catheterization on day 0. Each experiment included two unchallenged sentinel animals. Urine samples were collected as in Fig. 1A. Level of bacteriuria over time was determined as the number of bacteria per mL of urine measured by quantitative PCR. Thin lines represent individual animals and thick lines indicate geometric means within each group. Dotted lines indicate the lower limit of quantification (LLOQ). (B) Body temperature (°F) of cynomolgus macaques was measured by microchip and rectal thermometer. (C) Blood neutrophil count. (D) Serum Interleukin-1β (IL-1β), Interleukin-6 (IL-6) and Tumor Necrosis Factor-α (TNFα) levels measured over time. Dotted lines represent the reference range of values in healthy animals (IL-1β: 0 – 10.1 pg/mL; IL-6: 0 – 271.3 pg/mL and TNFα: 0 – 169 pg/mL). Thin lines represent individual animals and thick lines indicate geometric means within each group.

SUPPLEMENTAL FIGURE 2

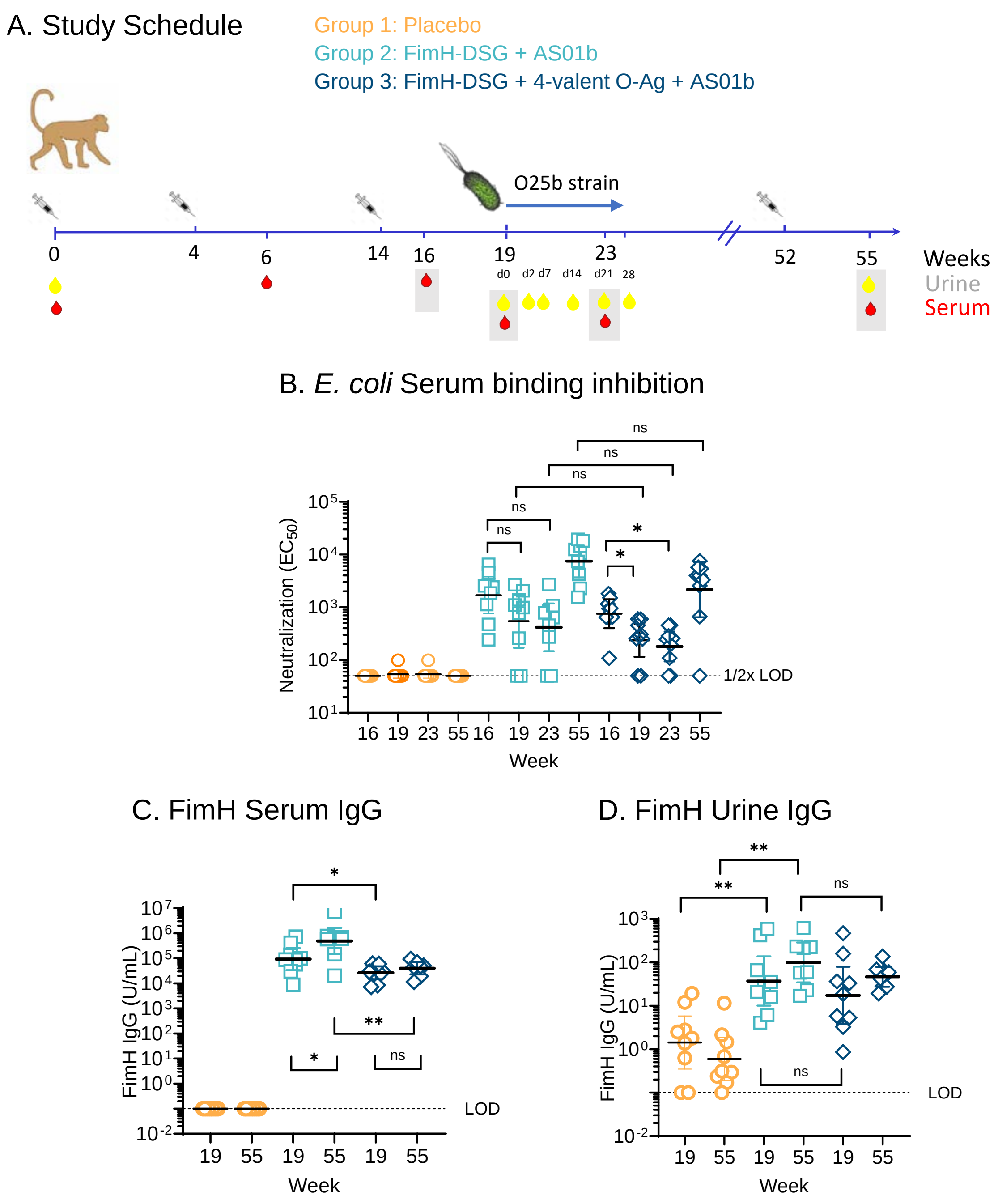

Fig. S2. (A) Dosing and post-challenge sampling schedule for vaccine and placebo control groups. Grey boxes highlight relevant timepoints. (B) Serum yeast mannan ligand binding inhibition (EC_{50}) titers at various pre-and post-challenge timepoints. (C) Serum anti-FimH IgG titers at time of challenge (wk19) and after fourth vaccine dose (wk55). (D) Urine anti-FimH IgG titers. Brackets denote significance comparisons for log transformed data: * $p < 0.05$, ** $p < 0.005$, ns – not significant. Dotted lines indicate assay limit of detection baseline values (LOD).

Table S1. Anti-FimH Serum IgG GMTs in Non-human Primates

Group	Placebo		FimH-DSG		FimH-DSG + 4-valent O-Antigen	
**Time point**	**PD2**	**PD3**	**PD2**	**PD3**	**PD2**	**PD3**
**GMT**	0.76	0.76	1965.69	4500.47	951.61	1457.49

GMT: Geometric mean titers; PD2 & 3: Post-dose 2 & 3

Table S2. *E. coli* Neutralization Assay IC_{50} GMTs for Non-human Primate Sera

Group	Placebo			FimH-DSG			FimH-DSG + 4-valent O-Antigen		
**Time point**	Pre-bleed	PD2	PD3	Pre-bleed	PD2	PD3	Pre-bleed	PD2	PD3
**GMT**	50	50	50	50	293.65	1698.39	50	480.12	756.45

GMT: Geometric mean titers; PD2 & 3: Post-dose 2 & 3

Table S3. O-antigen Serotype-specific Titers in Non-human Primates Vaccinated with FimH-DSG and 4-valent O-antigen

	O1a			O2			O6			O25b		
**Week**	pre	PD2	PD3	pre	PD2	PD3	pre	PD2	PD3	pre	PD2	PD3
**GMT**	3.8	2903.3	3446.4	4.3	5449.6	5696.6	5.1	2412.4	1686.8	2.6	3483.8	4028.5

GMT: Geometric mean titers: pre-immune week 0, week 6 (PD2) and week 16 (PD3)

Table S4. **Day of *E. coli* challenge antibody titers and D2/D7 post-challenge biomarker responses**

	NHP ID	Serum Neut titers (week 16)	Serum Neut titers (week 19)	Serum titers IgG (week 19)	Urine titers IgG (week 19)	Urine Day 2 post-challenge (wk19)			Urine Day 7 post-challenge (wk19)		
						Bacteriuria (qPCR)	IL-8 (pg/mL)	MPO (pg/mL)	Bacteriuria (qPCR)	IL-8 (pg/mL)	MPO (pg/mL)
FimH Group	AP423B	1128	259	57800.5	28.3	1.70E+06	18.1	269.0	2.50E+07	24.3	156.3
	CT341	243	50	8779.7	4.1	3.30E+06	22.1	608.2	1.40E+06	133.4	1190.0
	GA530N	2693	1389	727368.5	35.6	1.00E+02	2.6	156.3	2.30E+02	3.3	156.3
	CT436	2409	774	138734.8	66.3	3.60E+05	8.3	214.9	5.80E+06	25.4	245.0
	CT438	2548	1103	99025.1	21.1	1.60E+02	2.6	172.0	4.70E+02	4.8	156.3
	BF456I	6492	2685	418797.2	422.0	2.70E+02	4.8	318.9	1.00E+02	2.6	156.3
	CT522	4569	2068	86897.81	595.31	7.60E+02	47.6	NT*	3.60E+02	3.8	NT*
	F645RB	1857	987	89044.7	15.9	1.90E+04	4.9	156.3	1.00E+03	9.5	174.1
	JR20J	472	50	29749.2	6.2	1.00E+02	2.6	205.8	2.70E+02	2.6	156.3
FimH + O-Ag group	AM861H	1807	597	45957.6	18.8	1.00E+02	10.1	554.1	1.20E+02	4.6	156.3
	YS47G	620	284.9	61794.1	36.6	1.70E+05	9.8	200.7	5.50E+05	6.9	156.3
	GA950G	1156	250	8546.5	0.9	1.10E+02	10.6	178.0	1.00E+02	6.5	156.3
	CT302	651	50	24679.3	5.8	1.00E+02	2.6	173.1	1.00E+02	21.1	156.3
	GB52A	934	256	32681.5	5.3	2.30E+04	160.8	551.5	1.00E+02	28.5	156.3
	CT325	961	598	29148.6	33.3	1.00E+02	2.6	156.3	1.00E+02	51.1	156.3
	CT353	109	50	7293.5	3.3	4.60E+04	83.5	156.3	6.40E+05	25.7	156.3
	CT437	653	308	24304.3	470.9	5.90E+02	3.8	246.7	4.80E+05	4.4	156.3
	CT689	1512	455	59742.0	159.0	5.30E+03	7.9	228.7	1.30E+03	4.2	156.3
Placebo Group	SZ98H	50	50	0.1	0.6	5.60E+06	129.5	1727.7	1.80E+05	19.9	156.3
	AK55H	50	50	0.1	0.1	2.80E+05	41.9	252.7	8.40E+06	102.3	910.0
	CT469	50	50	0.1	12.1	1.50E+06	439.3	1489.4	4.10E+05	49.9	156.3
	BF925E	50	50	0.1	2.8	2.90E+06	14.7	287.3	1.80E+07	50.0	310.0
	BF923G	50	50	0.1	2.5	4.70E+05	19.4	173.6	1.90E+05	39.1	156.3
	CT414	50	50	0.1	19.3	5.10E+05	23.9	156.3	1.80E+07	26.0	156.3
	CT441	50	50	0.1	1.8	1.10E+06	22.5	310.8	4.40E+06	18.1	156.3
	CT339	50	50	0.1	1.4	2.10E+06	38.9	966.7	2.50E+07	21.2	185.0
	CU114	50	50	0.1	0.1	2.00E+06	285.1	917.7	2.30E+06	21.8	0.0

NT* - Not tested, insufficient sample volume

Fig S3. Correlation Analysis

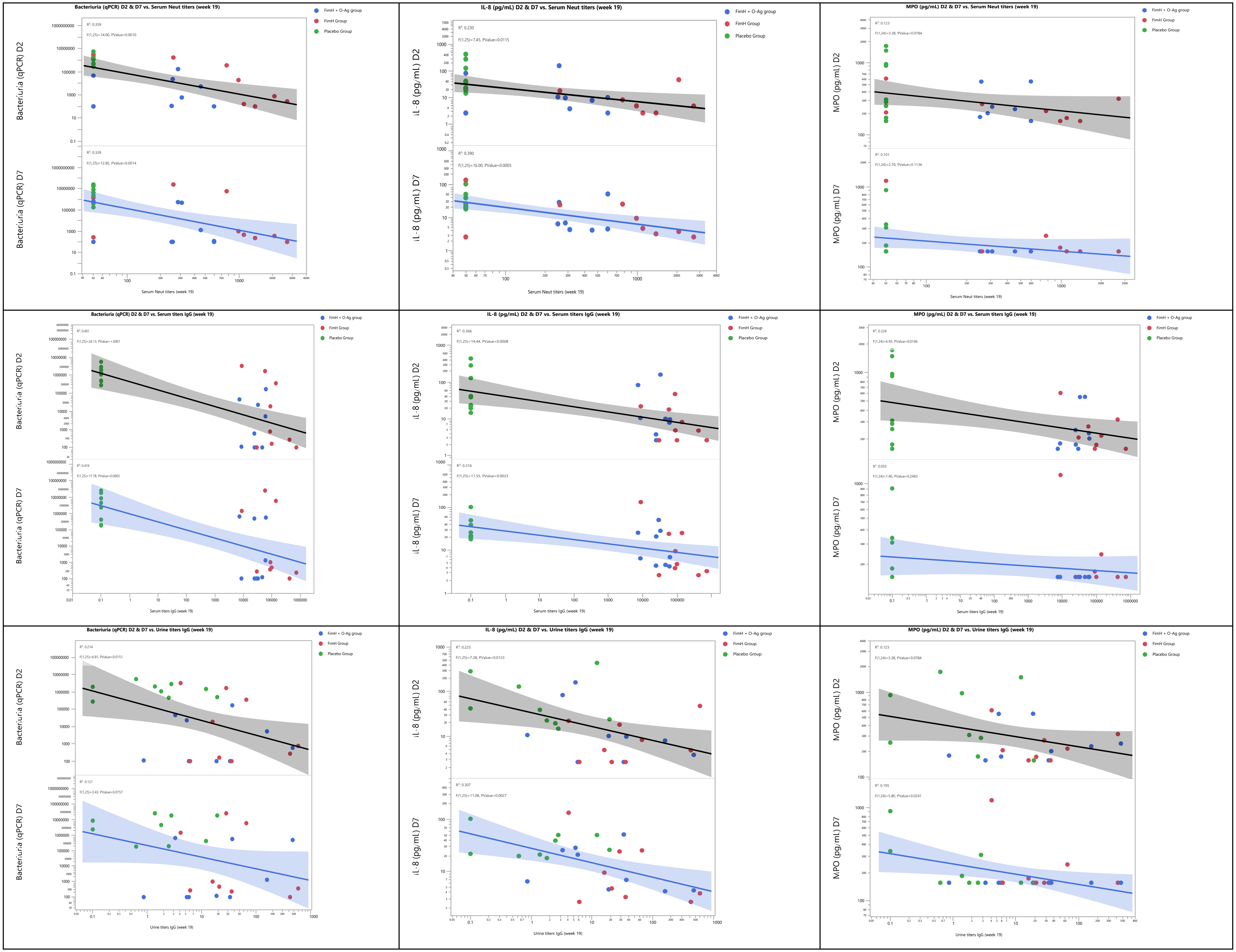

Fig. S3. Linear regressions of vaccine induced antibody and UTI biomarkers on day 2 and day 7 post-challenge. (top panel: serum neutralization titer vs. 3 UTI biomarkers; middle panel: serum IgG titer vs. 3 UTI biomarkers; bottom panel: urine IgG titer vs. 3 UTI biomarkers). R^2 values in the plots represent the square of the Pearson correlation coefficients.

Table S5. Correlation Summary

Titer	Response	*Correlation (r)	P-value
Serum Neut titers (week 19)	Bacteriuria (qPCR) D2	-0.6	0.0010
	IL-8 (pg/mL) D2	-0.48	0.0115
	MPO (pg/mL) D2	-0.35	0.0784
	Bacteriuria (qPCR) D7	-0.58	0.0014
	IL-8 (pg/mL) D7	-0.62	0.0005
	MPO (pg/mL) D7	-0.32	0.1136
Serum titers IgG (week 19)	Bacteriuria (qPCR) D2	-0.7	<0.0001
	IL-8 (pg/mL) D2	-0.61	0.0008
	MPO (pg/mL) D2	-0.47	0.0146
	Bacteriuria (qPCR) D7	-0.64	0.0003
	IL-8 (pg/mL) D7	-0.56	0.0023
	MPO (pg/mL) D7	-0.23	0.2483
Urine titers IgG (week 19)	Bacteriuria (qPCR) D2	-0.46	0.0151
	IL-8 (pg/mL) D2	-0.47	0.0123
	MPO (pg/mL) D2	-0.35	0.0784
	Bacteriuria (qPCR) D7	-0.35	0.0757
	IL-8 (pg/mL) D7	-0.55	0.0027
	MPO (pg/mL) D7	-0.44	0.0241

* Correlation (r) is based on log transformed antibody titers and biomarker responses. Significant correlation values are highlighted in red.

Table S5. Pearson correlation analysis values of associations between antibody titers at week 19 (day of challenge) and D2 and D7 post challenge biomarker responses. Plotted data are shown in supplemental Fig. S2. Source data are shown in Table S4, and the associated correlation plots in Fig S3.
